# Characterization of Sterilizing‐Grade Membranes/Prefilters and Application to mRNA‐LNP Process Development

**DOI:** 10.1002/bit.70089

**Published:** 2025-10-25

**Authors:** Kevork Oliver Messerian, Anton Zverev, Jack F. Kramarczyk, Andrew L. Zydney

**Affiliations:** ^1^ Department of Chemical Engineering The Pennsylvania State University, University Park Pennsylvania USA; ^2^ Moderna Inc. Cambridge Massachusetts USA

**Keywords:** lipid nanoparticle (LNP), mercury intrusion porosimetry, mRNA vaccine, prefiltration, sterile filtration

## Abstract

Sterile filtration is an essential step in the production of lipid nanoparticles (LNPs) used in mRNA vaccines and therapeutics. The overlap between the particle size of the LNPs (typically 50–200 nm) and the pore size of the sterilizing‐grade membranes (rated at 0.2 µm) complicates the design and operation of the sterile filtration process. The objective of this study was to characterize the pore size distribution, fouling behavior, and capacity of different sterilizing‐grade membranes and prefilters for LNP filtration. Mercury intrusion porosimetry revealed significant variability in pore size distributions across several sterilizing‐grade membranes despite their consistent 0.2 µm rating, with LNP filtration capacity strongly correlated with the experimentally observed mean pore size. Dual‐layer membranes, including the 0.8/0.2 µm Sartopore 2 XLG, significantly enhanced LNP filtration capacity and reduced fouling due to their integrated prefilter layer. Prefilter membranes with pore sizes in the 400–800 nm range provided the greatest enhancement in LNP filtration capacity. These findings highlight that for a given LNP formulation, the filtration capacity is strongly influenced by sterilizing‐grade filter selection and specifically by the membrane pore size distribution and morphology, which are generally not published by filter manufacturers.

## 1 Introduction

The application of sterile filtration for traditional biologics such as monoclonal antibodies and recombinant proteins is relatively straightforward due to the significant size difference (approximately an order of magnitude) between the product of interest and the 0.2 µm pore size of the sterilizing‐grade filters (Lutz et al. 2013). However, while all sterilizing‐grade filters are designed to effectively retain microorganisms and large particulates, their filtration capacity can vary significantly, particularly when processing larger biotherapeutics such as virus‐like particles and lipid nanoparticles (Messerian et al. 2023a). These performance differences are often attributed to the structural and material properties of the membranes, which directly affect their filtration performance (Na et al. 2022). Key factors such as pore size distribution, pore structure (asymmetric vs. symmetric), membrane chemistry (hydrophilicity vs. hydrophobicity), and other morphological characteristics (e.g., porosity, thickness, tortuosity, and pore interconnectivity) also play an essential role in determining the overall performance and efficiency of these filters.

Sterile filtration of large particle size therapeutics may result in low yields, limited filtration capacity, and operational challenges. These challenges generally arise from the overlap between the membrane pore size and the size of the particles, resulting in increased propensity for filter fouling. Note that the filtration behavior of these larger biotherapeutics can also depend on their deformability, with rigid particles (like most viral vectors) behaving differently than deformable liposomes and lipid nanoparticles.

One approach that can be used to increase the capacity of the sterile filter is to use an appropriate prefilter. The prefilter can be configured as a standalone filter placed upstream of the sterilizing‐grade filter or it can be part of an integrated dual‐layer device (commercially available from many filter manufacturers). The prefilter generally has a larger pore size, allowing it to remove larger foulants and thereby protect the sterilizing‐grade membrane (Griffiths et al. 2016). For example, the 0.8 µm prefilter of the dual‐layer Sartopore 2 XLG membrane has been previously demonstrated to increase the sterile filtration capacity of a mRNA‐LNP feed stream by approximately 2.5‐fold compared to the 0.2 µm sterilizing‐grade membrane alone (Messerian et al. 2022). Similarly, Wu et al. (2025) demonstrated that increasing the prefilter pore size from 0.45 to 0.8 µm or from 0.8 to 1.2 µm significantly enhanced the filtration capacity of mRNA‐LNPs through the 0.2 µm sterilizing‐grade Sartopore membrane. While these studies confirmed the critical role of the prefilter on LNP filtration performance, comprehensive pre‐filter screening studies with mRNA‐LNPs have not been published. Even greater increases in capacity were seen with a glycoconjugate vaccine drug substance using a 5 µm prefilter (Du et al. 2021). In this case, the large improvement in capacity was a direct result of the removal of trace quantities of very large aggregates ( > 1 µm in size) present in the drug substance. More recently, Kapila et al. (2025) demonstrated that dual‐layer, asymmetric membranes significantly outperform single‐layer, sterilizing‐grade membranes in the sterile filtration of nanoemulsions used to deliver highly hydrophobic drugs.

The objective of this study was to characterize the pore size distribution of several prefilter and sterilizing‐grade membranes, to evaluate their fouling behavior and capacity, and to identify design features that can increase the capacity during constant pressure filtration of LNPs. Data were obtained using several commercially available membranes with different pore sizes and chemistries, including asymmetric filters in which the membrane support structure can act as an integral prefilter. These results provide important insights into the role of the pore size distribution of both the prefilter and the sterilizing grade filter in determining the capacity during LNP filtration.

## 2 Materials and Methods

Constant transmembrane pressure (TMP) sterile filtration experiments were performed using the commercially available sterilizing‐grade membranes shown in Table 1, both with and without different prefilters (Table 2). This included both homogeneous (symmetric) membranes, in which the pore size was relatively uniform through the depth of the membrane, and asymmetric (anisotropic) membranes that have a significant pore size gradient. Initial experiments examined the sterile filters alone, with subsequent experiments examining the impact of different prefilters as well as the prefiltration mode (batch, inline, or as an integral structure in which the prefilter is placed directly on top of the sterile filter in a single device).

**Table 1:** Commercially available sterilizing‐grade membranes used in this study.

Membrane	Manufacturer	Advertised pore structure	Chemistry	Nominal pore size rating (µm)
Sterilux	Meissner	Homogeneous	PVDF	0.2
Durapore	MilliporeSigma	Homogeneous	PVDF	0.22
Stylux	Meissner	Asymmetric	PES	0.2
Express Plus	MilliporeSigma	Asymmetric	PES	0.22
Sartopore 2 XLG (0.2 µm)	Sartorius	Asymmetric	PES	0.2

Abbreviations: PES = polyethersulfone, PVDF = polyvinylidene fluoride.

**Table 2:** Commercially available prefilters used in this study.

Membrane	Manufacturer	Advertised pore structure	Chemistry	Nominal pore size rating (µm)
Sartopore 2 XLG	Sartorius	Asymmetric	PES	0.8
Sartobran P	Sartorius	Asymmetric	Cellulose acetate	0.45
Sartopore Platinum	Sartorius	Asymmetric	mPES	0.45
Sartopore 2 XLI	Sartorius	Asymmetric	PES	0.35
0.8 µm MCE	MilliporeSigma	Asymmetric	Mixed cellulose ester	0.8
0.45 µm Sterlitech	Sterlitech	Asymmetric	PES	0.45
0.45 µm Durapore	MilliporeSigma	Homogeneous	PVDF	0.45
0.65 µm Durapore	MilliporeSigma	Homogeneous	PVDF	0.65

Abbreviations: mPES = modified polyethersulfone, PES = polyethersulfone, PVDF = polyvinylidene fluoride.

Data were obtained with an mRNA‐LNP solution supplied by Moderna Inc. (Cambridge, MA). These mRNA‐LNPs, referred to simply as LNPs, have properties consistent with those described by Hassett et al. (2021) with mean LNP diameter between 100 and 150 nm, with a significant number of LNPs larger than 200 nm. The LNP concentration was on the order of ~10^12^ particles per mL (Brader et al. 2021). The LNPs were frozen and then thawed in a room temperature water bath before use in the filtration experiments.

Filter membranes were housed within an Amicon ultrafiltration cell providing 4.5 cm^2^ of filtration area; thus, a volumetric throughput of 100 L/m^2^ corresponds to filtration of 45 mL of the LNP suspension. For evaluation of individual layers of dual‐layer membranes, like the Sartopore 2 XLG, the two layers were easily separated by tweezers so that the sterilizing‐grade layer could be examined separately. This caused no damage to the membranes.

Before each experiment, filtration membranes were flushed with at least 100 L/m^2^ of deionized water, with the water permeability evaluated by measuring the filtrate flux at several TMP values to verify that the filter was undamaged and that there were no leaks. The filter/system was then primed with Tris‐sucrose formulation buffer (Messerian et al. 2022). Filtration experiments were carried out according to the methods described previously (Messerian et al. 2024). Briefly, the LNPs were loaded into a 500 mL stainless steel reservoir (CT series, SR‐TEK, UK) and pressurized with compressed air using a pressure regulator. TMP was continuously monitored using a Track‐it pressure logger (Monarch Instruments, 4103064), while the filtrate flux was evaluated by mass collection using an OHAUS Ranger 3000 scale. LNP concentrations were determined based on the mRNA content using UV absorbance at 230 nm evaluated with a Tecan microplate reader. All experiments were performed at room temperature (21 ± 2°C).

### 2.1 Mercury Intrusion Porosimetry

A Micromeritics AutoPore V Model 9620 (Norcross, GA) was used to measure the pore size distribution of the different sterile filters using mercury intrusion porosimetry (MIP) (Gribble et al. 2011). To ensure reproducibility, four clean (unused) 47‐mm or eight 25‐mm filter discs were cut and loaded into the penetrometer, providing at least 0.2–0.3 g of membrane. For dual‐layer filters, the layers were separated and individually characterized. Mercury was incrementally intruded into the sample, with the pressure increased in steps of 2–20 kPa, allowing for equilibration at each step. After testing, mercury was reclaimed under vacuum, and contaminated filters were disposed of as hazardous waste.

MIP was used to evaluate the pore volume distribution for the different sterilizing‐grade membranes and prefilters. In each case, the pore diameters were evaluated from the data at different test pressures based on the Young–Laplace equation (Tanis‐Kanbur et al. 2021):

(1) $$P=\frac{4\gamma \cos\left(\theta \right)}{d_{p}}$$

where γ is the interfacial surface tension, θ is the contact angle between the fluid and pore, and $d_{p}$ is the effective pore diameter. The pressure data were first converted to pore diameter using Equation 1. The contact angle for mercury was taken as 140° (Calvo et al. 1995) and the surface tension was 0.485 N/m at room temperature. While 140° is a commonly used approximation, the actual contact angle can vary depending on the membrane surface chemistry, and in particular the hydrophobicity; this effect was not considered in the analysis of the MIP data. The volume distributions were then converted to number distributions by dividing the incremental volume of intruded mercury by the volume of a pore, which was modeled as a straight cylinder with the pore length equal to the membrane thickness (as determined by SEM (Taylor et al. 2021)). Note that MIP does not distinguish between “dead‐end pores” (pore volume that does not penetrate entirely through the membrane) and actual through‐pores, and it provides no information on the size of the pore constrictions that will likely determine the retention characteristics of the membrane (i.e., the retention properties of an “hour‐glass” shaped pore will mainly depend on the size of the narrow “neck” in the hour glass). As such, MIP provides a simplified view that may not fully reflect functional performance.

### 2.2 Bubble Point Measurements

Bubble points measurements were performed following the procedure outlined in (Messerian et al. 2023b) to provide an estimate of the largest pore in each membrane (Hernandez 1996). Briefly, membranes were mounted in 25 mm holders, pre‐flushed with 100 L/m² of deionized water, and pressurized with nitrogen at ~7 kPa/min. The bubble point was recorded as the pressure at which the first bubble appeared in a submerged dip tube. Measurements were performed in triplicate and reported as mean ± standard deviation in Table 3.

**Table 3:** Characterization of the sterilizing‐grade membranes using MIP and bubble point measurements.

Membrane	Observed mean pore diameter (µm)^a^	Observed largest pore diameter (µm)^b^	Observed water permeability (LMH/psi)	Porosity^a^ (%)	Thickness (µm)
Sterilux	0.25	1.09 ± 0.01	410 ± 10	74	100^c^
Durapore	0.27	0.65 ± 0.01	460 ± 10	57	100^c^
Stylux	0.28	0.78 ± 0.02	1350 ± 50	76	100^c^
Express Plus	0.33	2.41 ± 0.03	1080 ± 40	76	140^c^
Sartopore 2 XLG (0.2 µm)	0.35	1.03 ± 0.06	690 ± 10	76	140^d^

^a^ Determined by MIP

^b^ Determined by bubble point measurements

^c^ Sourced from Taylor et al. (2021)

^d^ Determined by SEM following the methodology outlined in Taylor et al. (2021).

## 3 Results and Discussion

### 3.1 Pore Size Characterization of Sterilizing‐Grade Membranes

Characterization results obtained with the five sterilizing‐grade membranes are summarized in Table 3. Even though all the membranes have a similar nominal pore size rating (0.2 or 0.22 µm), the number‐weighted mean pore diameter measured by MIP ranged from a low of 0.25 µm for the Sterilux membrane to as high as 0.35 µm for the 0.2 µm layer of the Sartopore 2 XLG. The largest pore diameter, based on the measured bubble point, ranged from 0.65 µm for the Stylux to 2.4 µm for the Millipore Express Plus. These values are consistent with results reported by the manufacturers and in the literature (Taylor et al. 2021). The different properties of the membranes are further highlighted by the detailed pore size distributions shown in the Supporting Information (Figure S1). The PVDF membranes (Sterilux and Durapore) exhibit both the smallest mean pore diameter and the lowest water permeability (410–460 LMH/psi), which reflects their tighter pore structure compared to the asymmetric PES membranes, all of which showed significantly greater permeability. Among the PES membranes, the permeability does not appear to directly correlate to mean pore size. Notably, the 0.2 µm Sartopore 2 XLG has the largest mean pore size (0.35 µm), yet its permeability is approximately half that of the Stylux membrane, despite both having similar porosity. This discrepancy may be due to differences in skin layer thickness or internal architecture not captured by MIP. These findings highlight how membrane chemistry and internal structure, beyond nominal pore size, are crucial design features that must be considered when selecting membranes for specific filtration applications.

**Figure 1:**
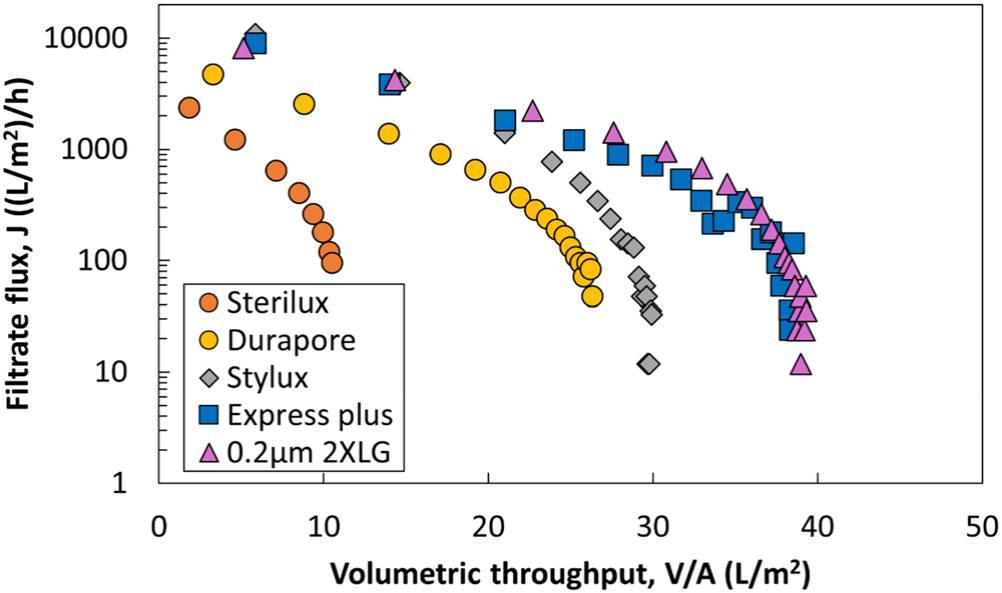
Flux profiles during sterile filtration of the LNP at a constant TMP of 20 psi through different sterilizing‐grade membranes. Highest capacity was achieved using the 0.2 µm layer of the Sartopore 2 XLG membranes while the lowest capacity was achieved using the Sterilux membrane.

Figure 1 shows experimental data for the flux during LNP filtration through each of these five membranes at a constant TMP of 20 psi. Although all membranes have similar nominal ratings (0.2 or 0.22 µm) and have high LNP recovery yields ( > 90%), their filtrate flux and capacity vary significantly, with up to three‐fold differences in filtration capacity (defined as the volumetric throughput at which the flux had declined to 10% of its initial value). Despite these differences, the flux decline profiles display a similar pattern of concavity, consistent with the complete pore blockage model reported previously (Messerian et al. 2022). Additionally, filtration capacities did not correlate well with the initial membrane water permeability or the bubble point. For example, despite having a substantially lower permeability than the Stylux membrane, the 0.2 µm layer of the Sartopore 2 XLG has the highest capacity (32 vs 22 L/m^2^ for Stylux).

**Figure 2:**
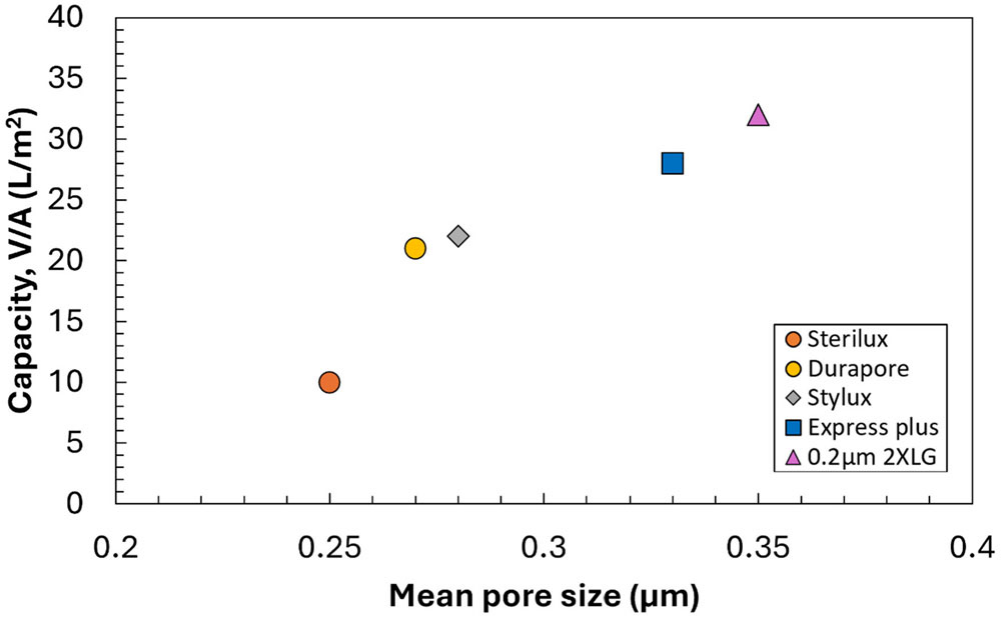
Filter capacity as a function of the number‐weighted mean pore diameter for the different sterilizing‐grade filters.

As shown in Figure 2, the capacity of the different sterilizing‐grade membranes is highly correlated with the mean pore diameter evaluated using MIP, with a simple linear regression fit giving *R* ^2^ = 0.86. The greatest capacity was obtained with the filter having the largest mean pore diameter (0.2 µm layer of the Sartopore 2 XLG) while the lowest capacity was obtained with the filter having the smallest mean pore diameter (Sterilux). This relationship is consistent with the expectation that smaller pores would be more easily blocked by the LNPs. Membranes with very small mean pore sizes, (i.e., more overlap with the size distribution of the LNPs) would likely show a disproportionately lower capacity, leading to the very low capacity observed with the Sterilux membrane. Additionally, the Durapore and Stylux membranes show similar capacity despite having different pore symmetry and membrane chemistry, indicating that these factors are not as impactful to LNP filtration capacity as is the mean pore size.

### 3.2 Effect of Prefilter Use

**Figure 3:**
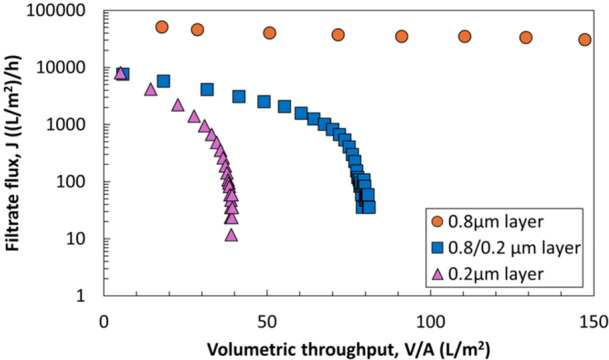
Filtrate flux as a function of the volumetric throughput for filtration of the LNP through the individual layers of the Sartopore 2 XLG at TMP = 20 psi. The 0.8 µm layer acts as a prefilter, protecting the downstream sterilizing layer from key foulants.

The filtration capacities of the single‐layer sterile filters (data in Figure 1) were all significantly smaller than the capacity of the dual‐layer Sartopore 2 XLG, which in previous LNP filtration studies using similar feed streams was shown to exhibit approximately two‐fold higher capacity (Griffiths et al. 2016). This observation suggests that the 0.8 µm prefilter provides significant capacity improvement by removing foulants responsible for limiting the capacity of the 0.2 µm layer. To investigate this phenomenon further, constant TMP filtration experiments were performed for the LNP with the individual layers of the Sartopore 2 XLG and compared to the dual‐layer configuration. As shown in Figure 3, the initial flux through the dual‐layer filter and the 0.2 µm layer are very similar, and much smaller than that for the 0.8 µm layer alone, indicating that the initial flux through the dual‐layer filter is dominated by the properties of the 0.2 µm layer. The flux decline through the 0.8 µm layer is negligible for the volume tested, indicating minimal fouling over the course of the filtration due to the large pore size relative to the foulants in this LNP formulation. The mean pore diameter of the 0.8 µm layer determined by MIP is 1.1 µm, which is three‐fold greater than that of the 0.2 µm layer. The capacity of the dual‐layer filter was approximately twice that of the 0.2 µm membrane alone, similar to previous findings with a similar LNP formulation (Messerian et al. 2022), demonstrating the effectiveness of the 0.8 µm prefilter.

### 3.3 Effect of Prefiltration Mode

**Figure 4:**
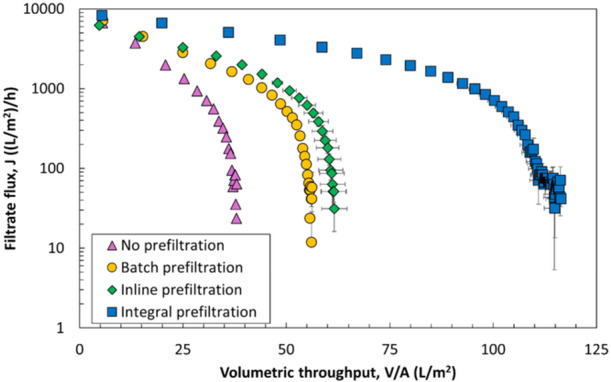
Filtration of LNPs through the two layers of the Sartopore 2 XLG membrane with different prefiltration modes (integral, inline, and batch) compared to no prefiltration (0.2 µm layer alone). Error bars are the standard deviation from repeat runs. Data were obtained at a TMP of 20 psi. Integral mode shows the highest capacity followed by the inline and batch modes.

To evaluate differences between various modes of prefiltration, experiments were performed using the 0.8 and 0.2 µm layers of the Sartopore 2 XLG in three configurations: (1) batch prefiltration, in which the feed is first filtered through the prefilter, with the permeate collected and then separately filtered through the sterilizing‐grade filter; (2) inline prefiltration, where the prefilter and sterilizing‐grade filter are placed in series in the same flow path; and (3) the integral (dual‐layer) configuration, in which both membranes are part of a single device. Results from these experiments are summarized in Figure 4. Although the addition of a prefilter significantly enhanced the filtration performance in all three modes, by far the best performance was obtained with the integral configuration. The inline configuration performed marginally better than the batch configuration, which provided only a 17% increase in capacity. Note that the difference in results for the integral configuration shown in Figures 3 and 4 is due to the different aliquot of LNP used for these experiments (which were subjected to different freeze‐thaw cycles).

These differences in fouling and capacity between configurations suggest that LNP foulants are not permanently removed by the prefilter and that LNP fouling may be influenced by time‐ and/or shear‐dependent mechanisms. The “lag time” between filtration through the prefilter and the sterilizing‐grade filter likely plays a critical role in determining the overall fouling behavior. This lag time varies from tens of minutes for the batch configuration, to several seconds for the inline configuration, to only milliseconds for the integral configuration. The lack of significant improvement in performance for the inline prefiltration compared to the batch prefiltration process suggests that LNP foulants may reaggregate fairly quickly (i.e., on a timescale shorter than several seconds). This behavior is consistent with previous findings (Griffiths et al. 2016), where sequential filtration of LNPs through two sterilizing‐grade filters using a batch setup failed to significantly improve filter capacity, suggesting that foulant removal during prefiltration is not permanent and complete. A similar discrepancy in performance between prefiltration modes was reported during virus filtration of monoclonal antibodies (mAbs), where the addition of a 5 µm Durapore prefilter significantly enhanced the flux and increased the capacity but only when used in the integral configuration. In that case, the improvement in performance was thought to be due to the time‐dependent self‐association of mAbs, with the mAbs re‐associating after passage through the prefilter when used in either the batch or inline configurations (Billups et al. 2022).

Alternatively, the integral configuration may help distribute flow more evenly across the sterilizing‐grade layer, which could reduce localized fouling and thereby improve overall filtration efficiency. The integral configuration could also have a preconditioning effect on the LNPs, with the shear forces involved in passage through the prefilter influencing the LNPs (or LNP aggregates) so that it is easier for them to pass through the sterilizing‐grade membrane. A preconditioning phenomenon has been previously reported during ultrafiltration of plasmid DNA (pDNA), with the pDNA effectively “pre‐stretched” by passing it through a larger pore size membrane (Li et al. 2015).

### 3.4 Effect of Membrane Asymmetry

The 0.2 µm layer of the Sartopore 2 XLG has a highly asymmetric pore structure, with the pore size decreasing from the upstream side to the downstream side (“skin‐side”). Asymmetric pore structures are intentionally designed to maximize permeability by reducing the thickness of the size‐selective skin (Tan and Rodrigue 2019). In addition, the more open pore structure in the support can increase filtration capacity by capturing foulants of different sizes, acting much like a depth filter (Li et al. 2009).

**Figure 5:**
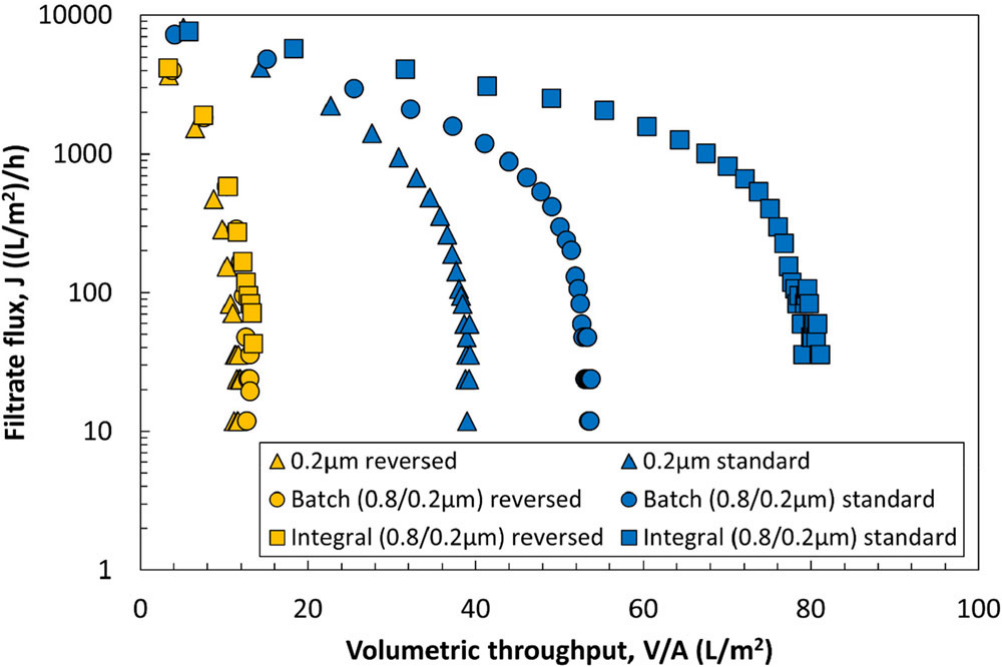
Effect of membrane orientation and prefiltration on the flux during constant TMP (20 psi) filtration of LNPs through the 0.2 µm layer of the Sartopore 2 XLG in both the standard (smaller pore size region facing downstream) and reverse (smaller pore size region facing upstream) orientations. Addition of a prefilter does not increase performance when the 0.2 µm layer is used in the reverse orientation.

To demonstrate the effectiveness of this design, additional prefiltration experiments were performed in which the orientation of the 0.2 µm layer was reversed so that the side with the smaller pore size was facing towards the feed flow; the 0.8 µm layer was used in its normal orientation. Filtration experiments were again conducted at a constant TMP of 20 psi, with results summarized in Figure 5. The capacities achieved in this reverse orientation are all smaller than those in the standard orientation. For the 0.2 µm layer alone, the initial flux in the reverse orientation is more than two‐fold smaller than in the standard orientation (3700 vs 8100 LMH) and the capacity was only 9 L/m^2^. In addition, the batch and integral prefiltration have minimal effect on the capacity of the 0.2 µm layer when used in the reverse orientation, demonstrating the critical role of the more open (larger pore size) upstream section of the asymmetric sterilizing‐grade membrane in reducing the extent of fouling. Note that the difference in results between Figures 4 and 5 is due to the use of a different aliquot of LNP for the experiments shown in Figure 4; Figures 3 and 5 were generated using the same LNP aliquots.

### 3.5 Effect of Prefilter Chemistry

**Figure 6:**
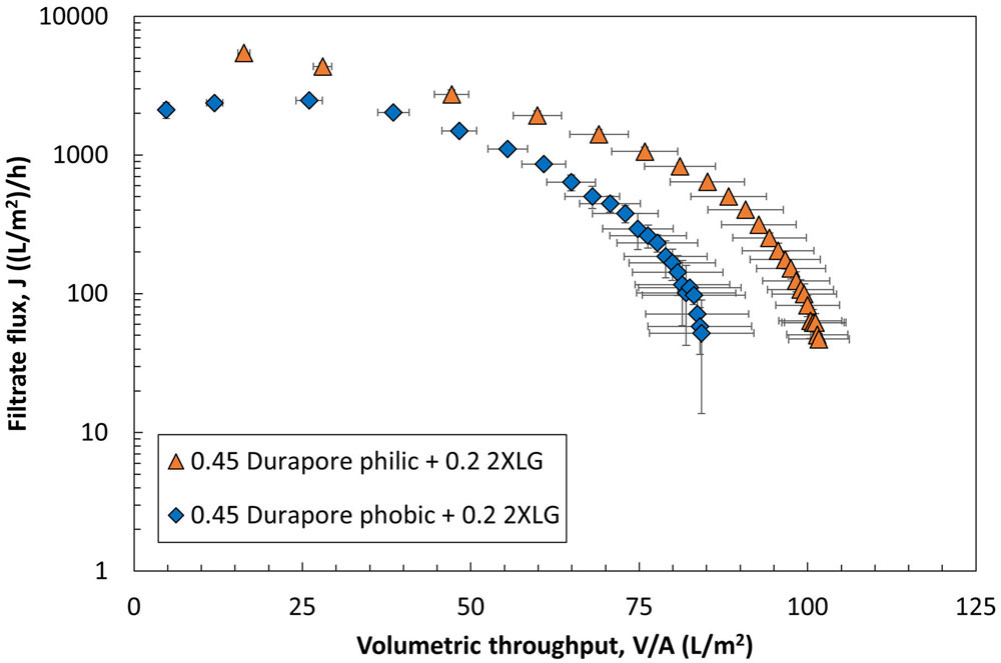
Flux profiles during filtration of the LNPs at constant TMP (20 psi) through dual‐layer filters formed using either a hydrophilic or a hydrophobic 0.45 µm Durapore prefilter stacked directly on top of the 0.2 µm layer of the Sartopore 2 XLG (integral prefiltration). Error bars are the standard deviations evaluated from repeat runs. Differences in flux and capacity between the two chemistries were relatively small, suggesting that the membrane chemistry of the prefilter does not play a major role in determining the prefilter effectiveness.

The filtration experiments performed with the different sterilizing‐grade membranes in Table 1 suggest that sterilizing‐grade membrane chemistry may not have a large effect on fouling and capacity for this LNP formulation. To further investigate the potential contributions from membrane chemistry, experiments were performed in which either a hydrophilic (HVLP02500) or a hydrophobic (HVHP02500) version of the 0.45 µm PVDF Durapore membrane was used as an integral prefilter for the 0.2 µm layer of the Sartopore 2 XLG (in its standard orientation). Although hydrophobic membranes are most commonly used for gas separations (Claramunt et al. 2021), the 0.45 µm pore size is sufficiently large that it is possible to wet the hydrophobic Durapore membrane with the aqueous LNP feed solution at relatively low TMP ( < 2 psi). The Durapore prefilters were selected because they are symmetric and have essentially identical pore size distributions (see Supporting Information, Figure S2). As shown in Figure 6, the initial flux through the dual‐layer membrane formed using the hydrophobic prefilter is approximately 2.5‐fold lower than that formed using the hydrophilic prefilter (2300 vs. 5500 LMH, respectively). Interestingly, the flux through the hydrophobic prefilter slightly increases during the initial stages of filtration before declining at higher throughput. Similar effects have been observed by Girones et al. (2005) when studying hydrophobic microsieve membranes, with this increase in flux caused by changes in the wetting of the hydrophobic pores. In this case, the change in wetting during the initial phase of the filtration may be due to interactions with the lipid components in the LNP feed. Overall, the dual‐layer membrane formed using the hydrophilic prefilter has a slightly higher capacity than that formed using the hydrophobic prefilter. However, the difference in performance between these membrane chemistries is relatively small (capacities within 20%), suggesting that the chemistry of the prefilter does not play a major role in the effectiveness of the prefiltration, at least for the LNP feed studied in this work.

### 3.6 Size of Foulants

**Figure 7:**
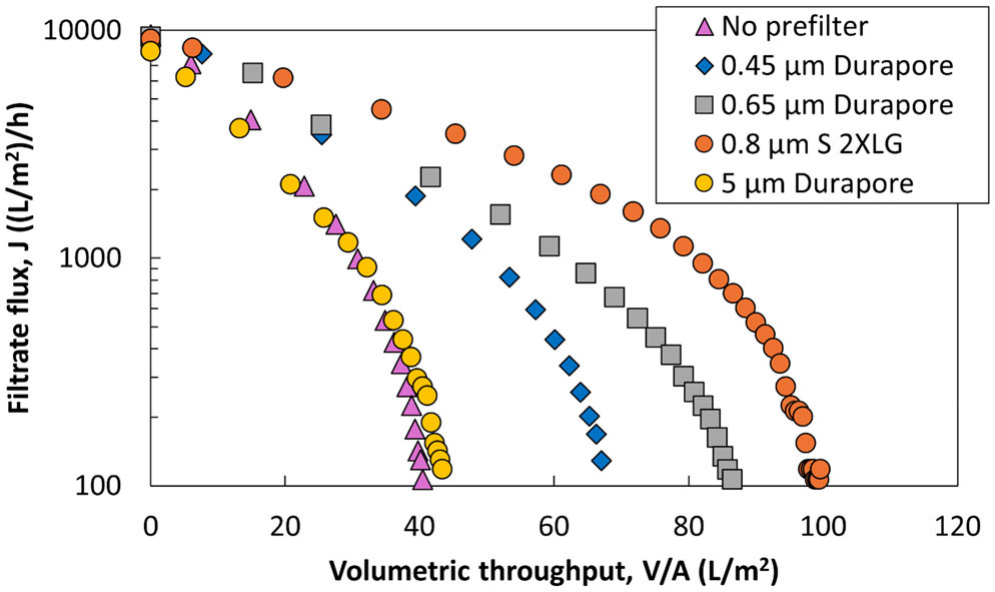
Filtrate flux as a function of volumetric throughput for dual‐layer (integral) filters formed by placing different pore size PVDF Durapore prefilters on top of the 0.2 µm layer of the Sartopore 2 XLG.

To investigate the impact of the size of the foulants in this LNP feed, several custom combination dual‐layer membranes were created by stacking hydrophilic PVDF Durapore prefilters of varying pore sizes directly on top of the 0.2 µm layer of the Sartopore 2 XLG in an integral configuration. The Durapore membranes were chosen for their homogeneous pore morphology and availability across a range of pore sizes. Figure 7 illustrates the performance of these dual‐layer membranes with prefilter nominal pore sizes ranging from 0.45 to 5 µm. The initial fluxes through all of the combination filters are very similar to that for the 0.2 µm layer of the Sartopore 2 XLG since the sterilizing grade layer is the primary contributor to the overall permeability. The filtration capacity increases with increasing prefilter pore size up to 0.8 µm, from 31 L/m² with no prefilter to 54 L/m² with the addition of a 0.45 µm prefilter, to 62 L/m² when using a 0.65 µm prefilter, and to 83 L/m² with the addition of a 0.8 µm prefilter (the upper layer in the commercial Sartopore 2 XLG). However, the 5 µm Durapore prefilter has almost no effect on the filtrate flux or capacity. These data strongly suggest that the foulants involved in LNP filtration are in the sub‐micron size range, being effectively removed by the 0.65 µm Durapore prefilters but showing minimal removal by the 5 µm Durapore prefilter. This behavior is in stark contrast to data obtained during sterile filtration of a glycoconjugate vaccine drug substance, where the addition of the same 5 µm Durapore prefilter led to more than a 100‐fold increase in capacity (Du et al. 2021).

### 3.7 Performance of Dual‐Layer Membranes

**Figure 8:**
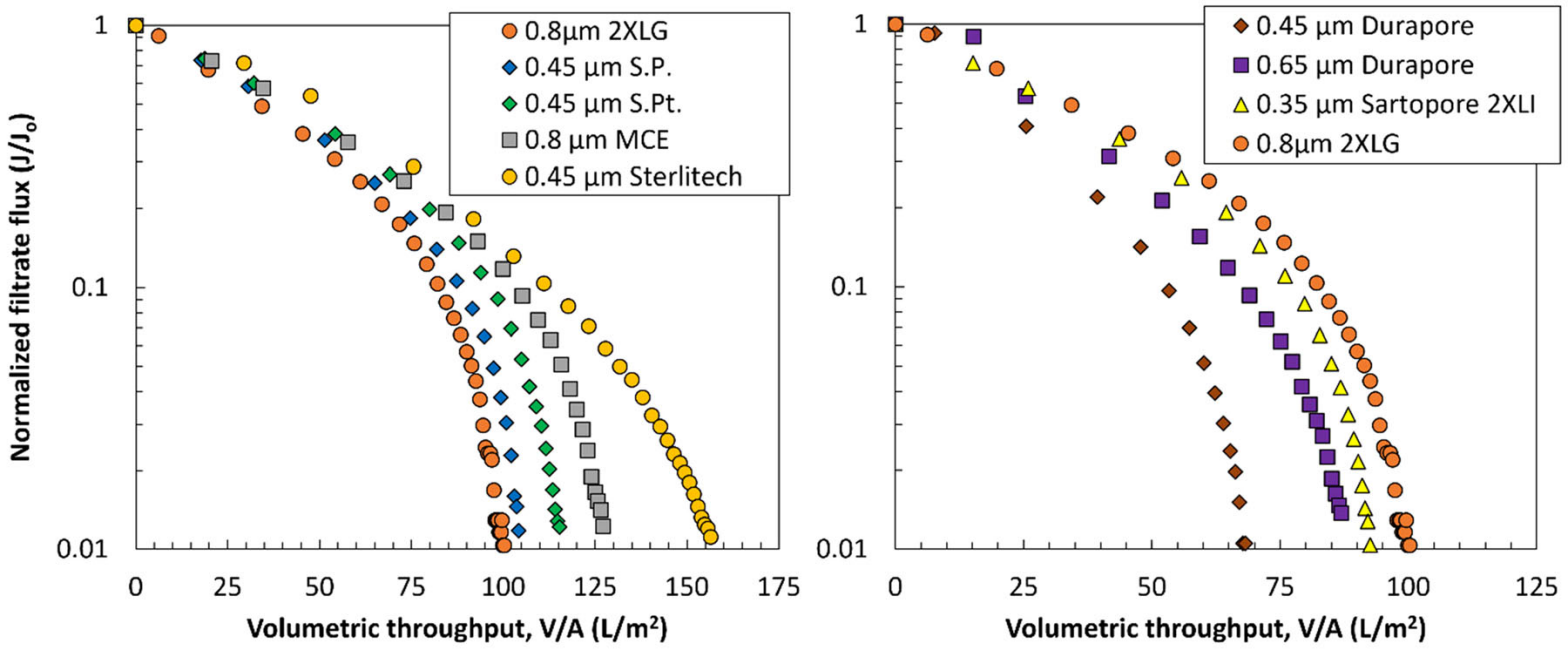
Normalized filtrate flux as a function of volumetric throughput for integral dual‐layer filters formed by placing different prefilters directly on top of the 0.2 µm layer of the Sartopore 2 XLG. The left panel presents a specific set of non‐commercially available dual‐layer membranes that outperform the capacity of the standard dual‐layer Sartopore 2 XLG membrane, while the right panel shows prefilters that have lower capacity than the dual‐layer Sartopore 2 XLG.

Further insights into the design of dual‐layer membranes for the sterile filtration of LNP were gained by testing various prefilters (Table 2) with different chemistries and pore size distributions (see Supporting Information, Figures S3 and S4) in combination with the 0.2 µm layer of the Sartopore 2 XLG. Similarly to the combinations evaluated in Figure 7, these custom, integral dual‐layer membrane combinations are not available commercially. As shown in the left panel of Figure 8, several of the evaluated custom dual‐layer configurations achieve a superior capacity compared to that of the dual‐layer Sartopore 2 XLG membrane (0.8 µm + 0.2 µm). The best performing prefilters included the 0.45 µm cellulose acetate (CA) prefilter sold as part of the Sartobran P (52307H‐47‐P‐B), the 0.45 µm polyethersulfone (PES) layer of the Sartopore Platinum (54907H‐47‐B), a 0.8 µm mixed cellulose ester membrane from MilliporeSigma (AAWG04700), and a 0.45 µm PES membrane from Sterlitech (PES4525100). These high‐performing prefilters span a range of membrane chemistries, again suggesting that it is the pore size and/or morphology, not the chemistry itself, that is critical in determining the performance of these novel dual‐layer filters. There were also many dual‐layer configurations evaluated that had worse performance than that of the dual‐layer Sartopore 2 XLG membrane, with several of these shown in the right panel of Figure 8. In particular, the relatively poor performance of the 0.35 µm prefilter (Sartopore 2 XLI) may well be due to its small pore size compared to the rest of the prefilters.

**Figure 9:**
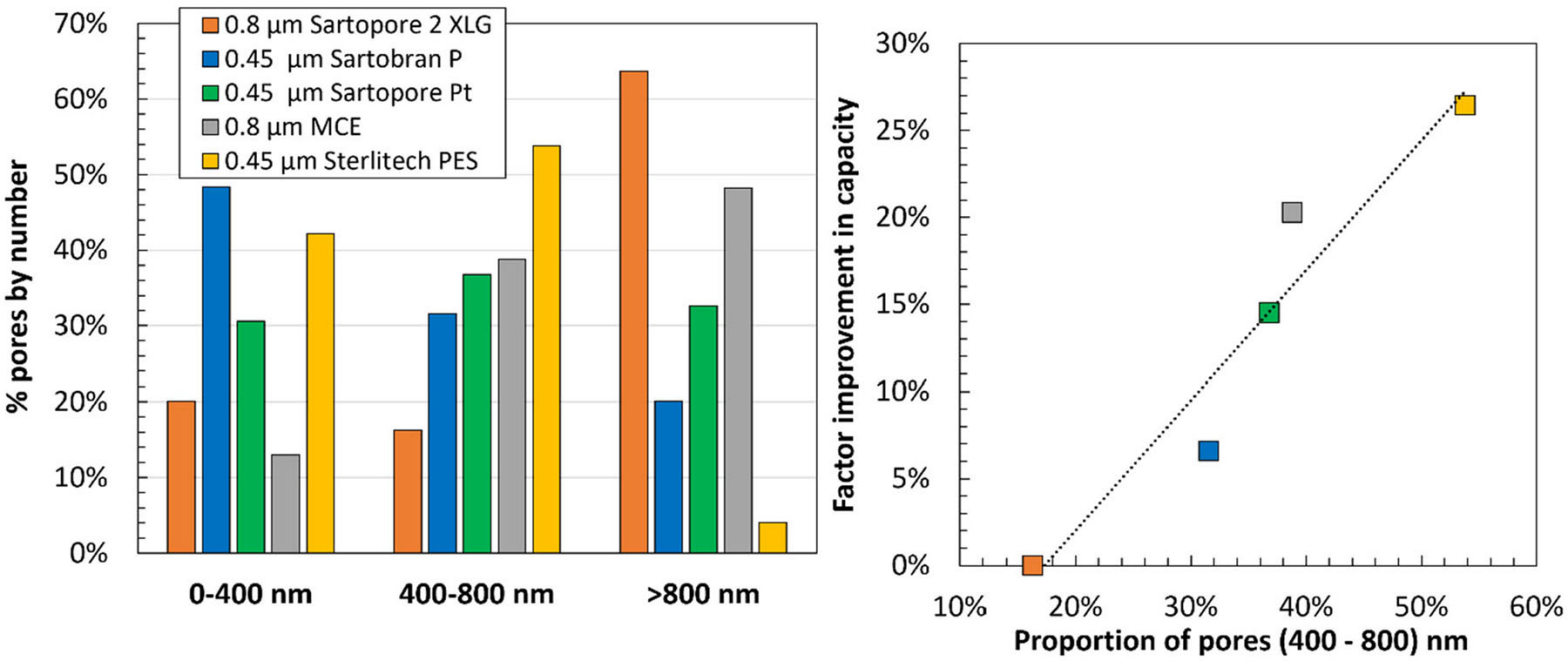
Number pore size distribution of the different prefilters binned into three pore sizes as determined by MIP (left panel). Right panel shows correlation between the improvement in filtration capacity and the percent of pores in the 400–800 nm range.

The detailed pore size distributions of the best‐performing prefilters were evaluated by MIP. In each case, the number distribution of pores was evaluated by considering three size bins: pores smaller than 400 nm, pores between 400 and 800 nm in size, and pores larger than 800 nm. The left panel of Figure 9 presents the percentage of pores within each size range for the different prefilters. All the prefilters have broad pore size distributions, with significant numbers of pores below 400 nm as well as a significant number of pores above 800 nm. For example, the 0.8 µm layer of the Sartopore 2 XLG has approximately 20% of its pores (based on the number distribution) smaller than 400 nm and over 60% of its pores larger than 800 nm. Conversely, the 0.45 µm cellulose acetate layer from the Sartobran P has a distribution skewed towards smaller pore sizes, with nearly 50% of its pores falling below 400 nm and only 20% exceeding 800 nm.

The prefilters that show superior performance are consistently found to have a higher percentage of pores within the 400–800 nm range, which is the approximate size range previously identified as being critical for effective prefiltration of this LNP formulation (Figure 7). This behavior is examined more closely in the right panel of Figure 9, which shows the improvement in capacity (defined as the relative increase in the capacity for the dual‐layer filter compared to that for the dual‐layer Sartopore 2 XLG) plotted against the percentage of pores in the 400–800 nm size range. The data show a high degree of correlation suggesting that the pore size distribution of the prefilter is a key factor determining the prefilter performance. Other metrics including porosity, median pore size, and the percentage of pores in broader or adjacent size ranges were also evaluated but did not show meaningful correlation with capacity. Note that this correlation does not hold for all prefilters; the Sartopore 2 XLI shows relatively poor performance even though 47% of its pores are in the 400–800 nm range (Supporting Information, Figure S4), likely due to the relatively high percentage of very small pores in this prefilter (38% of the pores are less than 400 nm in size).

### 3.8 Implications for Industrial LNP Filtration

Interestingly, despite differences in membrane chemistry, supplier, and configuration, the fouling behavior of the sterilizing‐grade membranes is largely consistent. In all cases, except when the Sartobran P was used as a prefilter, the data fit well to the complete pore blockage model (Messerian et al. 2022), suggesting that fouling for the mRNA‐LNP formulations examined in this work is due primarily to the deposition of larger LNP foulants onto membrane pores. Despite sharing the same fouling mechanism, substantial differences in capacity are observed across the tested membranes, driven by structural factors such as pore size distribution, layer orientation, and configuration. These results imply that membranes originally optimized for protein‐based biologics may not be well suited for other feed streams, including large particle size therapeutics like LNPs. In particular, the greater increase in capacity provided by prefilters enriched in 400–800 nm pores suggests that achieving a closer match between the size of the foulant and the size of the pores in the prefilter is key to reducing the rate of fouling. This highlights a new design space for membrane manufacturers, focusing on fine‐tuning sub‐micron pore size distributions to accommodate the unique filtration behaviors of large particle size therapeutics. Future studies will be required to demonstrate whether this behavior also holds for more rigid particles like viral vectors.

## 4 Conclusions

The results obtained in this study provide important insights into the fouling behavior and capacity of sterilizing‐grade filters during the sterile filtration of LNPs at constant TMP. All the sterilizing‐grade filters show qualitatively similar patterns of flux decline, suggesting that the underlying pore blockage fouling mechanism is the same for these filters. Pore size distributions of various sterilizing‐grade membranes and prefilters were analyzed using MIP, revealing significant variability in pore size even for sterilizing‐grade membranes with similar nominal sizes (0.2 or 0.22 µm). Filter capacity for the different sterilizing‐grade membranes is strongly correlated with the mean pore size, with the larger pore size membranes showing less fouling and higher capacity.

Much higher filtration capacities are obtained using dual‐layer filters, with the use of appropriate prefilters significantly reducing the fouling of a 0.2 µm sterilizing‐grade membrane (asymmetric PES, Sartopore 2 XLG). Interestingly, the effectiveness of the prefilter is strongly dependent on the mode of prefiltration, with the integral configuration achieving the highest capacity compared to either inline or batch prefiltration. The origin of the improved performance with the integral configuration is likely due to minimizing the re‐aggregation of LNP, possibly in combination with a preconditioning of the LNP that enhances their passage through the pores in the sterilizing‐grade membrane. Prefiltration also has no significant effect when the asymmetric sterilizing‐grade membrane is placed in a reverse orientation, indicating that the asymmetric pore structure of the 0.2 µm layer is critical to its capacity. Furthermore, MIP revealed that prefilters having a higher percentage of pores in the 400–800 nm range, when paired with the 0.2 µm layer of the Sartopore 2 XLG filter, provides the greatest enhancement in the filtration capacity of LNPs. These results clearly demonstrate the importance of the pore size distribution of the prefilter relative to that of the key foulants in the LNP feed. Experiments investigating the impact of varying prefilter chemistry and pore size revealed that pore size is the more significant factor, suggesting that membrane chemistry does not play a major role in LNP filtration performance.

## Author Contributions

**Kevork Oliver Messerian:** data curation, formal analysis, investigation, and writing – original draft. **Anton Zverev:** methodology, resources, and writing – review and editing. **Jack F. Kramarczyk:** funding acquisition, conceptualization, methodology, resources, and writing – review and editing. **Andrew L. Zydney:** conceptualization, funding, supervision, writing – review and editing.

## Conflicts of Interest

K.O.M, A.Z. and J.F.K. are employees of and shareholders in Moderna Inc. A.L.Z. declares no competing financial interest.

## Supporting information


**Figure S1:** Pore size distributions of the different sterilizing‐grade filters used in Figures 1 and 2 plotted as the incremental intrusion of mercury vs pore diameter. **Figure S2:** Pore size distributions of the 0.45 µm hydrophilic and hydrophobic Durapore membranes plotted as the number of pores vs pore diameter. Both membranes have mean pore size of 0.56 µm with a thickness of 100 µm. **Figure S3:** Pore size distributions of the different prefilters used in Figure 8 plotted as the number of pores vs pore diameter. The largest capacity was achieved with the 0.45 µm PES prefilter from Sterlitech which had a mean pore diameter of 0.49 µm. **Figure S4:** Pore size distributions of the different prefilters used in Figure 8 plotted as the number of pores vs pore diameter.

## Data Availability

The data that support the findings of this study are available from the corresponding author upon reasonable request.
