# Supplementary material for: Characterization of Sterilizing‐Grade Membranes/Prefilters and Application to mRNA‐LNP Process Development
Source: Biotechnol Bioeng. 2025 Oct 25;123(1):164–73. doi: 10.1002/bit.70089 (PMC12699132; doi:10.1002/bit.70089)
Supplement: Supplementary file 1 — Figure S1: Pore size distributions of the different sterilizing‐grade filters used in Figures 1 and 2 plotted as the incremental intrusion of mercury vs pore diameter. Figure S2: Pore size distributions of the 0.45 µm hydrophilic and hydrophobic Durapore membranes plotted as the number of pores vs pore diameter. Both membranes have mean pore size of 0.56 µm with a thickness of 100 µm. Figure S3: Pore size distributions of the different prefilters used in Figure 8 plotted as the number of pores vs pore diameter. The largest capacity was achieved with the 0.45 µm PES prefilter from Sterlitech which had a mean pore diameter of 0.49 µm. Figure S4: Pore size distributions of the different prefilters used in Figure 8 plotted as the number of pores vs pore diameter. [file BIT-123-164-s001.docx]

# **Supplemental Information**

# Characterization of sterilizing-grade membranes / prefilters and application to mRNA-LNP process development

*Kevork Oliver Messerian*[^a^](#a),[^b^](#b)*, Anton Zverev*[^b^](#b)*, Jack F. Kramarczyk*[^b^](#b) *and Andrew L. Zydney*[^a^](#a)[*](#Corresponding)

^a^Department of Chemical Engineering

The Pennsylvania State University

University Park, PA 16802

^b^ 325 Binney Street

Moderna, Inc.

Cambridge, MA 02142

Communication concerning this manuscript should be addressed to:

Andrew Zydney, Department of Chemical Engineering,

The Pennsylvania State University, University Park, PA 16802

Phone: 814-863-7113, Fax: 814-865-7846

E-mail: [zydney@engr.psu.edu](mailto:zydney@engr.psu.edu)

Figures S1 to S4 show the mercury intrusion porosimetry (MIP) results for the sterilizing grade filters and prefilters analyzed in this study. The homogenous membranes (e.g., the Durapore membranes in Figure S2) show narrower pore size distributions than the asymmetric membranes, with many fewer pores with diameter greater than 1 µm as expected. The different prefilters (e.g., the 0.8 µm layer of the Sartopore 2 XLG in Figure S3 show very broad pore size distributions with pores as large as 2 µm in diameter, consistent with the asymmetric structure of these membranes. Note that this method neglects the underlying structure of the asymmetric membranes in which the size-selective pores in the dense “skin” layer only penetrate a small distance into the filter.


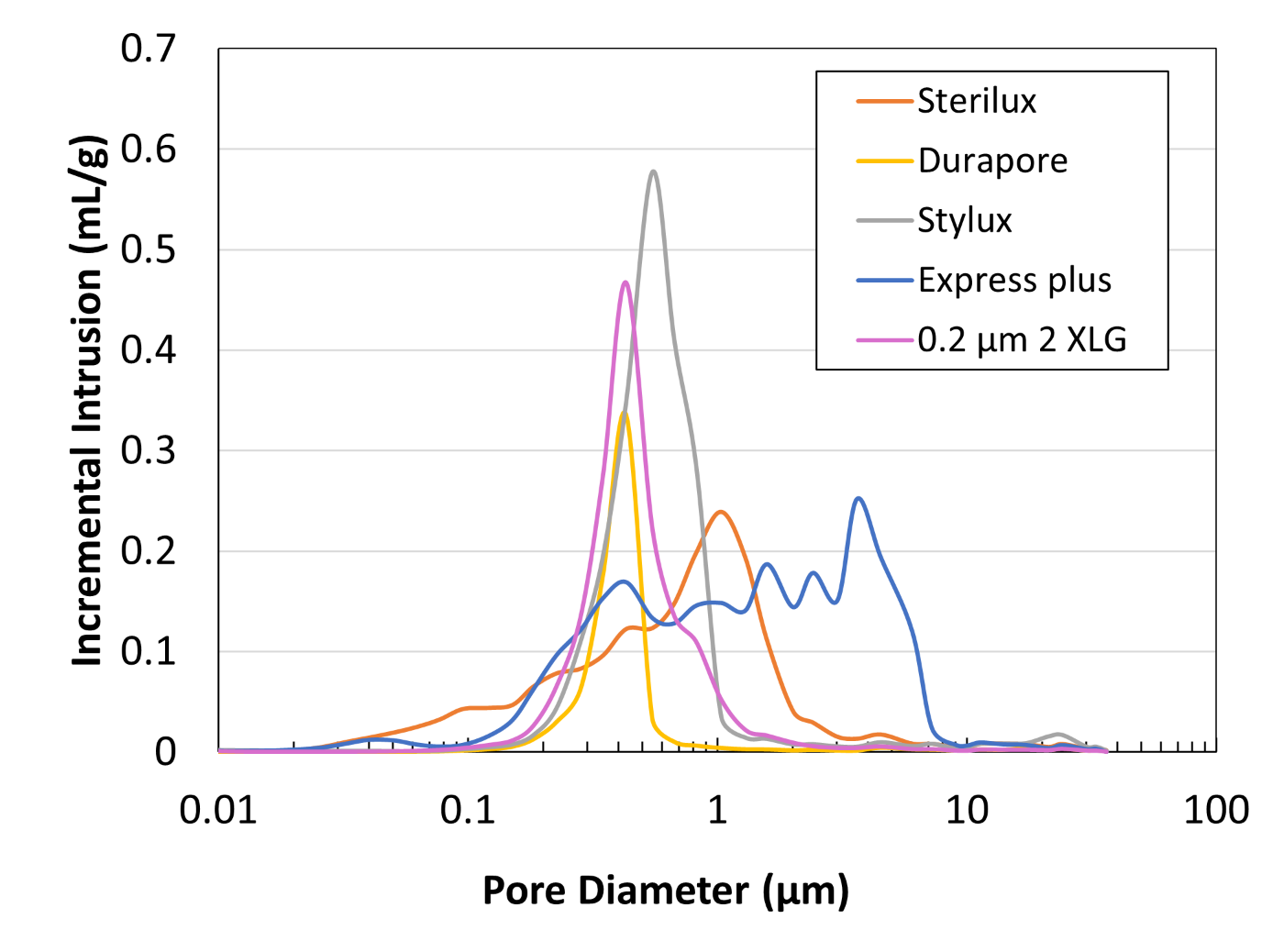


Figure S1: Pore size distributions of the different sterilizing grade filters used in Figures 1 and 2 plotted as the incremental intrusion of Mercury vs pore diameter.


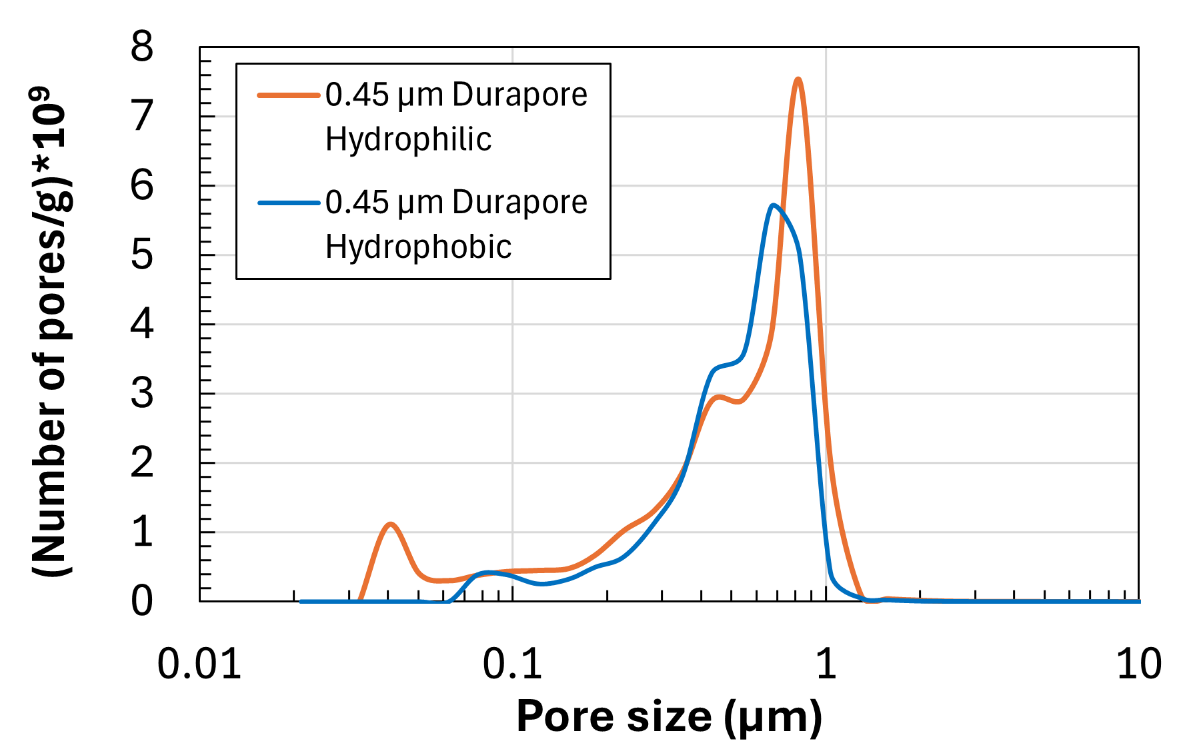


Figure S2: Pore size distributions of the 0.45 µm hydrophilic and hydrophobic Durapore membranes plotted as the number of pores vs pore diameter. Both membranes have mean pore size of 0.56 µm with a thickness of 100 µm.


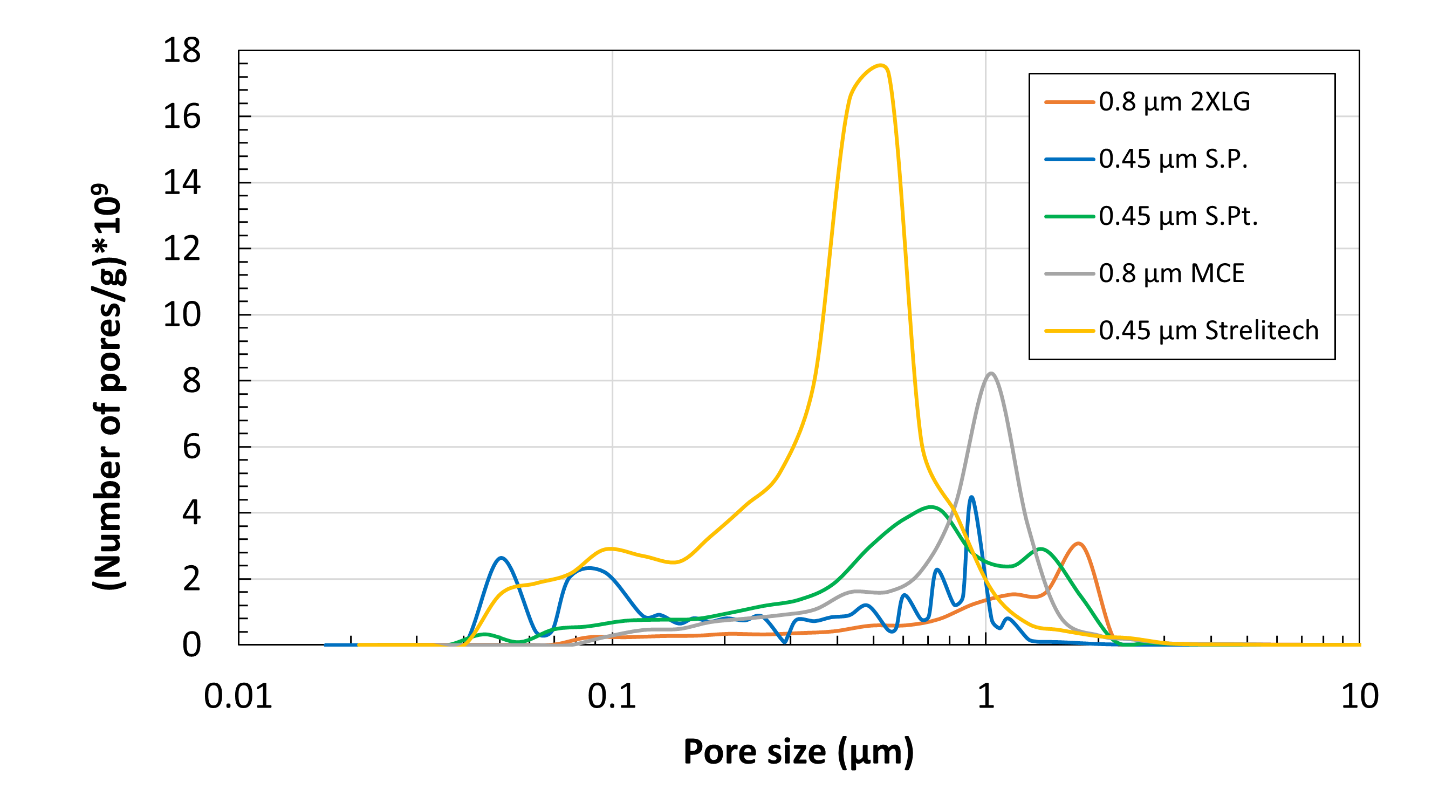


Figure S3: Pore size distributions of the different prefilters used in Figure 8 plotted as the number of pores vs pore diameter. The largest capacity was achieved with the 0.45 µm PES prefilter from Sterlitech which had a mean pore diameter of 0.49 µm.

*
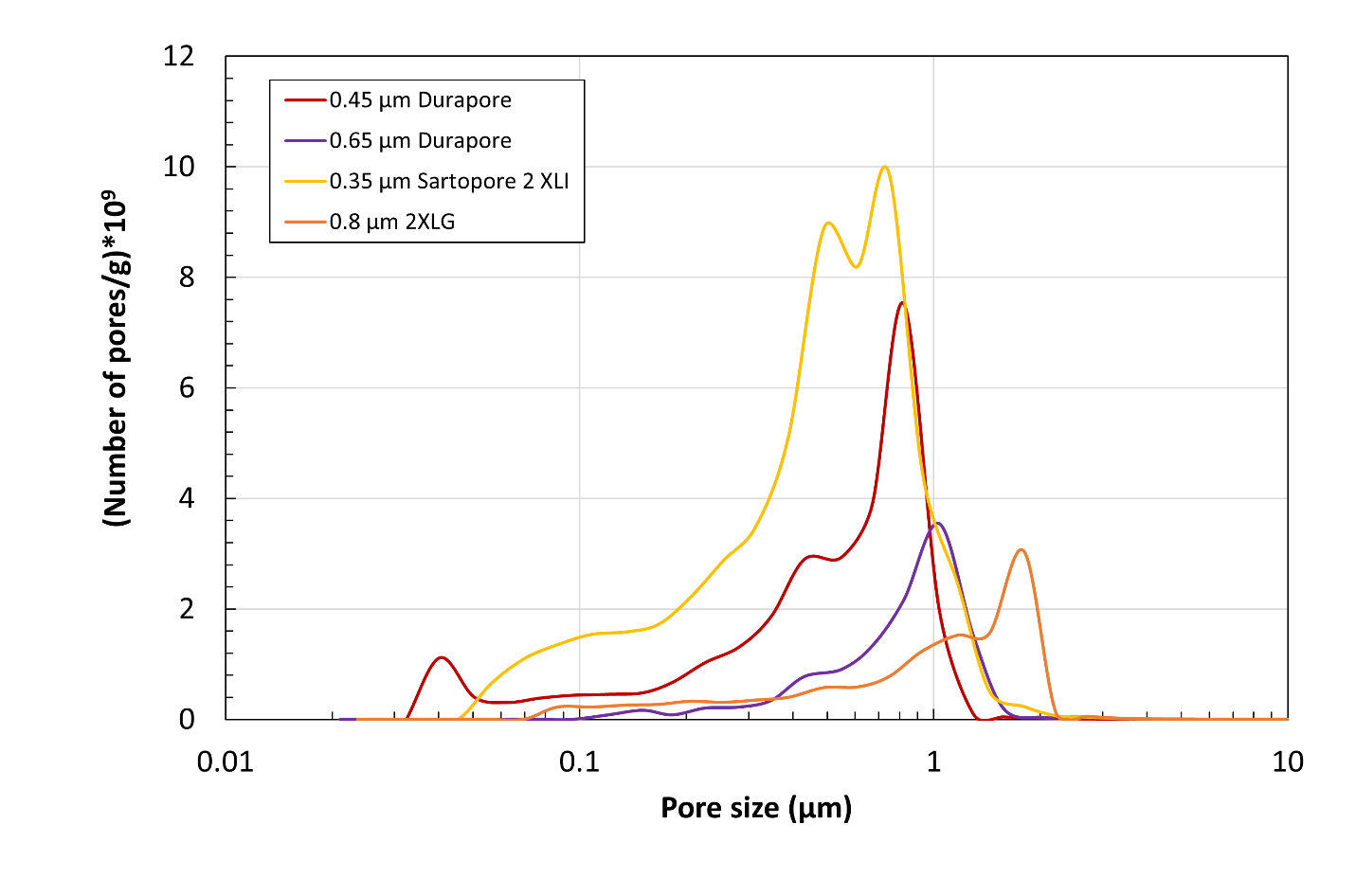
*

Figure S4: Pore size distributions of the different prefilters used in Figure 8 plotted as the number of pores vs pore diameter.
